# A longitudinal qualitative study of clinical nurses caring for hospitalized adults during the first fifteen months of COVID-19: lessons in professional survival and leadership

**DOI:** 10.1186/s12912-025-03628-2

**Published:** 2025-08-18

**Authors:** Elizabeth M. Norman, Mildred Ortu Kowalski, Roxanne Sabatini, Carol S. Jones, Wendy Silverstein

**Affiliations:** 1 New York University, New York, NY USA; 2 Morristown Medical Center, 100 Madison Avenue, Morristown, New Jersey, 07960 USA; 3 Chilton Medical Center, 97 West Parkway, Pompton Plains New Jersey, 07444 USA; 4 Morristown Medical Center, Talent Acquisition, 100 Madison Avenue, Morristown, New Jersey, 07960 USA; 5 Morristown Medical Center, Morristown, New Jersey, 07960 USA; 6 8 South Brookwood Drive, Montclair, NJ 07042 USA

**Keywords:** Longitudinal qualitative research, COVID-19, Camaraderie, Professional self-image, Professional disaster strategies, Leadership support, Kantian philosophy

## Abstract

**Background:**

The goal of this study was to record and analyze the initial and lingering thoughts and reactions that 41 clinical nurses experienced while caring for individuals hospitalized with COVID-19 during the initial April 2020 surge and over the next 15 months while vaccines and effective treatments became available. The sample represented a diverse group of nurses at one American health care institution in terms of gender identity, age, ethnic group, years of experience, clinical specialty area, and hospital shift worked. At the time of this April surge, nurses were exposed to a virus with no standard treatments and high mortality rates. Using a longitudinal qualitative design, the team hoped to answer questions about what compelled these nurses to continue to come to work? And what leadership lessons about identity, communication, camaraderie can be learned from this unplanned crisis?

**Methods:**

A longitudinal design was chosen to capture the changes in nurses’ thoughts about themselves as professionals and their clinical work during COVID-19 after the World Health Organization (WHO) declared COVID-19 a global pandemic on March 11, 2020 (Cucinotta and Vanelli, Acta Biomed 91(1):157–60, 2020 [1]). The research team wondered how a large American suburban medical center staff was dealing with this virus and if there were changing patterns in the experiences and thoughts of clinical nurses as the virus evolved in 2020–2021. We applied the 18th Century philosophy of Immanuel Kant writings on moral thinking to examine the nurses’ thoughts of continuing their clinical work during this time. Using a classic Western philosopher seemed relevant given the worldwide nature of the pandemic which affected everyone, not just health care workers. Interview questions from the literature and research team experiences were developed; an additional file provides details of the interview schedule in English (See Additional file 1 for the interview schedule). Forty-one RNs agreed to participate; each nurse agreed to three interviews over three phases. The first phase occurred from April through November 2020 with 41 RNs participating. Thirty-five RNs from the original 41 returned for a second interview between November 2020 through April 2021. In the final phase the same 35 RNs took part in a final interview between April 2021 and July 2021. We asked the same questions to all nurses during each phase. Similar quotations were grouped into 42 codes, then like codes were collected into five themes: Exposure risk, Professional Self-Image, Communication, Community Reaction and Finding an Emotional Balance. These themes provided a context for describing the ethos of nurses working with pandemic patients.

**Results:**

These nurses put the needs of the greater community before their personal desires. They came to work every day because they believed that they had essential knowledge and skills to help in the pandemic. Despite the realization that many of their patients would die, the nurses adapted their skills within strict isolation protocols. Their greatest stress was infecting their families, a misfortune that did not happen. Effective leadership and the camaraderie that developed among the nurses and other staff sustained them during this time. Interview quotations seemed to mirror interviews conducted by one team researcher on military nurses who served in Vietnam (1962–1975). Comparing these citations suggests that the personal danger, inability to save people and an overwhelming number of patients that taxed resources were similar to veteran and civilian nurses.

**Conclusion:**

The 35 clinical nurses in this study worked through the initial fifteen months of COVID-19 pandemic in America. Kant’s idea of duty for the greater good over personal needs provided an explanation and rationale why the 35 clinical nurses remained in clinical work. Reviewing the transcripts indicated that there seemed to be parallels between nurses’ clinical experiences in the Vietnam War and the COVID-19. Their experience indicated that planning for future disasters is imprecise. Knowledge gleaned from this study suggests that a combination of external factors and personal assets should provide the support for nurses and other health care workers in future plagues.

**Trial registration:**

This study was registered retrospectively on ClinicalTrials.gov NCT06012539 on August 23, 2023.

**Supplementary Information:**

The online version contains supplementary material available at 10.1186/s12912-025-03628-2.

## Background

In early 2020 as COVID-19 admissions and deaths skyrocketed, the Center for Disease Control (CDC) published an article advising American hospital leaders to prepare for a virus that would strain bed capacity, supplies and personnel [2]. The World Health Organization (WHO) warned countries that viral transmission knowledge would continue to evolve but mortality and morbidity could be reduced with early diagnosis and quality care [3]. China, Thailand, Japan, and Korea were the first nations to report cases [4]. The CDC reported the first confirmed American case on January 18th 2020 [4].

The study site was a 700 + bed non-profit, tertiary suburban medical center in Northern New Jersey, USA, where 1600 registered nurses worked. Medical center administrators prepared an internal playbook on treatments and isolation policies that were communicated to staff on all units [5]. These administrators regularly reported census, mortality and morbidity rates to State leaders. The figures from the first eight weeks of the pandemic at the medical center during March-April 2020 illustrated the widespread seriousness of the virus: 879 COVID-19 admissions with 232 deaths and 647 patients surviving [6].

On March 5th 2020, physicians at the study site admitted the first adult with a COVID-19 diagnosis. Four weeks later the hospital had 287 patients with COVID-19 and 95 deaths [6]. Clinical nurses had to follow untested protocols to help them care for very sick patients. We began the study interviews in April 2020 with the goal of obtaining nurses real-time thoughts and behaviors at the onset of the pandemic; at a later time as treatments and vaccines were being developed and final interviews a year later as the virus became entrenched in daily life. The two aims of the study were to examine nurses’ thoughts about caring for patients with COVID-19 over 15 months and to explore why most of these professionals remained at their positions while other nurses left, often citing COVID-19 as a reason [7, 8]. This new knowledge might support both clinical nurses and nurse managers when faced with future unsafe or challenging times (e.g. pandemics, disasters).

## Methods

### Study design

A longitudinal design was chosen with the aim of capturing the changes in nurses’ thoughts about themselves as professionals and their clinical work as the COVID-19 pandemic shattered everyday living (emn, mok, rs, cj, ws). Across the study phases, we incorporated questions about the nurses’ coping strategies and the impact of those strategies on their decisions to remain at work.

Two team members conducted all interviews (rs, mok). The first interviews occurred from June through November 2020 and focused on the nurses’ ability to provide care during the March-April surge. The second interviews occurred from December 2020 through April 2021. We reviewed their comments from the first interview and added questions about the new COVID-19 vaccines and the impact on their work and lives. The third interviews, which took place from April 2021 until August 2021, focused on their thoughts about COVID-19 becoming part of their daily routine. We also talked about what they learned about working in a disaster, and advice they would share with other nurses about working during the pandemic.

### Sample

Atlantic Health System Institutional Review Board (IRB) approved how the sample was obtained and protected. The team aimed for a diverse group of nurses who would present a range of responses to our questions. Participants were registered nurses who provided direct care to adult patients with COVID-19 for at least 12 h between March 23-April 23, 2020. After IRB approval, the team obtained a list of nurses who provided this direct care and then randomized the names based on the usual clinical area worked. This list was confidential and kept on a secure website. Nurses on the list were approached in the order of randomization. The team contacted eighty-five nurses (mok). Forty-four did not respond to multiple calls or emails from team members (rs, mok, ws). The sample was reviewed for demographic variables: gender, years of experience, age, day shift/night shift, clinical area worked and race/ethnic background. The sample was complete when there were consenting nurses representing each demographic variable. Names were not linked to transcripts, rather numbers were used. We did obtain a diverse sample of 41 nurses (See Table 1). Participants agreed to take part in three interviews which would take place over a year. However, six RNs did not participate in Phases Two and Three. The six who did not participate either left to work at another facility (*n* = 1), stated they were too busy (*n* = 1) or never responded to at least three attempts at contact (*n* = 4). Thirty-five nurses remained in the study and completed the three interviews.

**Table 1:** Demographic information

Characteristic	*N* = 41 (%)
Gender, n (%)	
Male	11 (27)
Female	30 (73)
Age, mean (SD)	42.78 (13.92)
Race/Ethnicity, n (%)	
White	26 (63)
Black/African American	2 (5)
Hispanic/Latino	1 (2)
Asian	11 (27)
Unspecified	1 (2)
Years of Experience	
N	38
mean (SD)	14.75 (12.89)
median (IQR)	9.5 (3.38, 27)
Shift Worked, n (%)	
Days (7am to 7pm)	22 (54)
Nights (7pm to 7am)	14 (34)
Other	5 (12)
Clinical Area, n (%)	
Ambulatory	2 (5)
Cardiac	5 (12)
Emergency Department	5 (12)
Intensive Care Medical	3 (7)
Intensive Care Surgical	6 (14)
Maternal Child	3 (7)
Oncology	1(2)
Perioperative Services	4 (10)
Step Down	6 (14)
Surgical Unit	6 (14)

### Ethical considerations

Atlantic Health System Institutional Review Board (IRB) approved the study’s written consent form and research protocol (IRB clinical trial number 1605198). This study was registered retrospectively with clinicaltrial.gov (NCT06012539) on August 23, 2023. We obtained informed written consent from all participants prior to starting the interviews.

We assumed every nurse in the study would experience some level of distress recounting prior pandemic events while still caring for patients with COVID-19. During the consent process, investigators emphasized that the interviews could be ended at any time, an option no one requested. Written consent for an interview, recording, and consent to publish were obtained from all participants and stored in a secure website prior to the individual’s first interview (mok, rs, ws). At the end of each interview, the interviewer gave each nurse a list of names and phone numbers of hospital, national and veterans mental health resources. At subsequent interviews we asked about any resources they used and how effective each nurse felt this support was.

### Study credibility

Two team members (rs, mok) conducted all the interviews for consistency. These repeated conversations over fifteen months helped build rapport with the participants. Other measures used to improve study credibility included a triangulation of sources, peer review, prolonged time in the field and member checking. We triangulated each nurse’s three interviews and field notes to enhance accuracy. For peer review, our team met every Friday afternoon via Teams software to review codes, issues and progress. At the start of Phases Two and Three, the interviewers and nurses reviewed the previous conversations for agreement, corrections and amplifications.

The interviews took place in a hospital private office where nurses could sit six feet apart to avoid wearing masks during the conversation. One nurse participant chose to remain masked during a Phase One interview; the other nurses and the interviewers remained maskless. After each interview, the room was sanitized. The average interview was 45 min with a range from 30 to 90 min. We audio recorded all the interviews and stored them on a secure website. A professional transcriptionist transcribed all recordings.

We used ATLAS.ti v9 software to code the transcripts [9]. No artificial intelligence was used; coding was done by the researchers (emn, mok, rs, cj, ws). An additional file shows codes and themes in more detail [see Additional file 2]. One team member, who did not interview (emn), read and recoded eight transcripts from each study phase to check the reliability of the initial coding, a process called intercoder agreement (ICA), twenty-four transcripts in total. She used the percentage of agreement of applied codes between herself and another interviewer to determine an ICA. The results were: Phase One overall agreement was 90% for 38 codes with a range of 86–94%; Phase Two overall agreement was 87% for 41 codes with a range of 82–94%; Phase Three overall agreement was 92% for 42 codes and a range of 90–97%. The combined ICA for all phases was 87%. Experts in these methods recommend an agreement of at least 80% for credibility [10].

All interviews have been edited for concision and clarity by the authors.

## Results

The medical center’s morbidity and mortality figures from the first eight weeks of the pandemic in March-April 2020 illustrated the catastrophe, highlighted by the life-threatening nature of the virus: 879 COVID-19 admissions with 232 deaths and 647 patients surviving [6]. Study participants spoke of the changes that occurred as the hospital census and mortality rates rose. Emergency department (ED) staff practiced putting on protective gear (PPE) and moving infectious patients. Facility teams erected barriers to create a segregated bed annex in the Medical Intensive Care Unit (MICU) with its own medication dispensing cabinet. Nurse leaders created a COVID-19 resource book to be distributed to every unit. Pharmacists reviewed drug protocols, and an engineering team evaluated a nearby decommissioned hospital about a mile away as a location for hospice patients.

Starting March 11^th,^ 2020, no visitors were allowed into the hospital buildings [11]. Two weeks later, the Governor directed all hospital directors in the state to close surgical units and restrict surgeries only to urgent non-elective cases like brain aneurysms [12]. Hospital administrators designated critical care units and medical units for patients with the virus. Nurses were reassigned to COVID-19 units to reinforce staffing [11].

In March 2020, the staff began to use a new argot with words like “Donner,” a team member who checked that Personal Protective Equipment (PPE) was put on correctly, and a “Doffer,” a team member who checked that PPE was removed in the correct order [11]. Nursing leaders posted daily staff assignments in text messages, emails, phone calls or on a list of names posted at the entrance to a parking garage. Workers drilled holes through hallway walls into patient rooms. Nurses could then administer intravenous medications, hydration, and nutrition through these holes to the bedside without entering the room. Hospital carpenters cut openings and inserted glass into patient room doors [11]. Outside of each room a sticky mat was placed to capture particles on shoes.

Despite these preparations, COVID-19 patients overwhelmed the hospital staff. The volume and mortality rate were first apparent in the Emergency Department (ED) [5]. In the ED, staff registered patients, brought oxygen to the cars as patients waited to be brought into the hospital. Nurses began to warn drivers not to pull away and disconnect the portable oxygen tanks sitting at the curb. Panicked patients and families needed assurance:

One person erratically cut in front of the other cars and parked sideways. The driver got out and started screaming that if we did not take care of his father, the man would die. We explained that we would get to him as soon as possible. Families were out of the cars screaming. We got it worked out, but it was hard. I was trying to put myself in his place. I knew the son could not come in. I also knew that if the older man comes in, he will probably not see him again. (Participant #38)

### Phase one findings

#### Theme one: exposure risk

The fear of catching the virus and bringing it home was ever-present. Participant #34 recalled a ventilator’s contaminated effluent spilling while turning a patient. He kept checking himself for symptoms. After ten days, he told himself he was ‘good’ but shaken as he returned to work.

Others tried to balance their fears of bringing the virus home by creating a specific routine:

I didn’t stay in a hotel because my kids had asked me not to. But when I got home, I didn’t see them much either, because I was so afraid to get them sick. I ate separately and slept separately. I got naked the moment I got onto my porch and then into a shower. When I got out, I’d cover my face, give them an elbow bump, and say, ‘Mom loves you.’ I slept on the floor in my bedroom for two months, so that I didn’t even breathe near my husband in the bed. (Participant #12)

A year after the pandemic began, the American Nurses Association released news that more than 500 nurses had died from the virus; *Beckers Hospital Review* journal reported that this first year, 32 per cent of all known healthcare worker deaths from COVID-19 were nurses [13, 14]. Five of the 41 nurses in this study contracted COVID-19 during the data collection phase. They quarantined at home. All returned to the hospital and continued to care for viral patients, saying that experiencing the symptoms made them more understanding of patient’s fears.

#### Theme two: professional image

When asked what they thought about themselves as professional nurses prior to the pandemic, they mentioned “integrity,” “proficiency,” “advocacy” and “compassion.” Experienced nurses said the years of service, especially with AIDS patients, helped them weather the COVID-19 crisis. They did not consider leaving their positions. As Participant #1 said about her 40 years as an RN said,

I have a long homecare and hospice history. So, I was able to process the death and dying aspect, probably easier than some nurses who were [from] telemetry, cardiac, you know what I mean?

Still, even experienced nurses could not do their work as they had:

The first time I had all my PPE on with my glasses, everything was fogged. I tried to draw blood. I felt so frustrated, I walked out of the room and started crying. (Participant #26)

Inexperienced nurses, who received their RN licenses less than a year before March 2020, had difficulty dealing with the soaring admissions. Participant #11, who graduated from school 18 months prior, said,

Are you going to be taking direct care of patients or were you going to be assisting? It felt like you got thrown to the wolves. I didn’t know if I was ready to deal with something like that professionally.

Caring for people who would not recover no matter what the nurses did was anathema to their beliefs as caregivers. Participant #24 nurse remembered watching her dying patient when she heard the attending physician say, “Don’t look at me like that. We’re already doing everything we can. You have to accept that.”

Every nurse said the pandemic changed the way they practiced. They realized that it took 30 min to don their PPE. A hospital-wide practice limited time in isolation rooms, care was clustered, a change from pre-pandemic times when each nurse determined how much time they would need. Now, they could not get to know their patients. They had baths, feeding and other tasks to do in less than an hour. These constraints were difficult for staff who were used to touching, talking to patients and getting their work finished. They had to become more efficient.

Good moments happened, especially when patients went home. As discharged patients climbed into wheelchairs, unit staff lined the hallways and clapped. When they neared the hospital entrance, the hospital speakers played the Beatles’ song, “Here Comes the Sun©” [15]. One nurse said the music also sent a message “We are making progress. We are winning a ‘war’.” (Participant #38).

#### Theme three: communication

Staff wrote technical notes on patient windows: 2:25 AM, 4/18, 1100 psi, peak 31 to connect people inside and outside the rooms. Because families could not visit, cell phones calls became vital. Nurses restructured their time to become familiar with their assigned patient’s charts before speaking with families. Even with this information, these calls could be challenging. One participant (#10) recalled that after intubations, she would speak with families and say,

We’re finished. It went well. They’re comfortable. Then they’d [the families] would say, ‘How long are they going to be on a ventilator?’ We had no idea. But you don’t want to say, ‘It could be 2–3 weeks.’

Once the iPads^®^ with visual software arrived, families could see their relatives. The devices delivered relief to families who could then see and speak with patients, but they also brought heartbreak especially when families cried and said their final goodbyes.

“You get a small glimpse that they once had a life and a family,” Participant #10.

The nurses needed to stay in contact and assure their own families they were all right. They wondered how their children were doing with their remote learning. Any problems with groceries? Are the monthly bills paid? Their families worried too, as Participant #38 learned:

I did not realize that my caring for patients with COVID-19 was so hard on my family. My daughter wrote a paper about COVID-19 and me working. One evening we were sitting outside talking about things. I wanted to talk about what would happen if I got COVID-19, if I got sick, or worse. My children said, we “don’t want to hear it.” For them, speaking about it made it real.

#### Theme four: community reaction

The outpouring of local community support made the staff realize people appreciated what the staff was doing. Strangers delivered restaurant meals, pizza, Girl Scout cookies^®^, and homemade meals. On St. Patricks’ Day 2020, revelers drove parade cars and fire trucks while bagpipers marched around the traffic circle at the front of the hospital. Staff stood at the windows and waved.

One day, the staff noticed a man standing outside the ED windows with his hand over his heart holding a sign:

Thank you all in emergency for saving my wife’s life. I love you all.

But the community was not of one mind. Participant #12 went to a local store to buy Easter candy encountered the sour with the sweet:

I went to get baskets for my kids. A woman at the counter said that this virus was all a farce; like a Democrat hoax or something. I lost myself. I screamed at the woman. I told her that I had watched several people die that week. How could she ever say such a thing? She turned around and yelled back, ‘Thank you for your service.’ I was so mad and shouted back, ‘How dare you say this.’ I just wanted to leave. When I got to the counter to pay for the baskets, the young man at the register said, ‘Thank you for saying something, because I have to deal with that all day and my mom doesn’t want me to work here.’ I replied to him, ‘Yeah, I get it. If I were your mom, I wouldn’t want you to work here either.’ He was like, 16.

#### Theme five: finding an emotional balance

The demands of working began to take a toll. Nurses talked about finding some activity, like a thirty-minute workout, which made them feel better. If they didn’t take time for themselves, they felt they were just sleeping and working.

We didn’t know how long it was going to stay really bad. You couldn’t continue running six days a week. (Participant #24)

Many stopped reading the newspapers or listening to the news, instead choosing meditation, yoga, journal writing, or a long shower. They took walks at night to decompress or during the day to as participant #24 said, “to add some brightness to this dark time.”

Strong friendships developed among the staff; a camaraderie shaped by a virus and daily sorrow.

We had a different bond. We solved things that came out of a nightmare. Problems I don’t think my family could really understand. (Participant #27)

One day, a novice nurse watched her first patient die and felt she needed to get away. Her preceptor took over the postmortem care which allowed this novice to regroup and return to work.

Another participant (#17) remembers calling a coworker on her way home. They talked about their difficult shift and the nurse said,

I went home and sobbed; but you know, I went back to work the next day.

Technology provided another way to communicate and receive support. Some nurses formed text messaging groups. Others formed or joined social media groups. A group set up and had Zoom meetings every Tuesday. Without those talks and texts,

You felt alone. Once you realize that you’re not the only one that’s felt this way, it’s comforting. (Participant #40)

### Phase two findings

We conducted second interviews with 35 RNs from the original 41. These interviews began during a surge of 380 COVID-19 admissions and 59 deaths from mid-November to mid-December 2020. This phase ended four months later, in April 2021, when the four-week census was 54 admissions and five deaths [6]. The fluctuating virus rates, and the introduction of vaccines led to the decision to place two COVID-19 patients in the same semi-private hospital room. On May 26, 2020, the Governor re-opened the operating rooms for all surgeries [16].

The new vaccines provided a bright spot. As the nurses received vaccinations, their worries about infecting their families lessened. Those nurses who volunteered to vaccinate the public at a shopping mall during an off day got a surprise. Participant #38 remembered

Believe it or not, vaccinating people helped me a lot, because people were so appreciative. I had quite a few people crying when they got their vaccine. When everybody said, ‘Thank you for what you’re doing,’ I said, “It takes a village.”

But the reality of caring for COVID-19 patients seemed relentless.

We’ve been doing it for a while now. You take the report in the morning and they’re all the same: COVID-19 pneumonia, on a ventilator, on these IV drips. It’s all supportive care. (Participant #15)

#### Theme one: exposure risk

In 2020, most spent their Thanksgiving, Christmas, Hanukkah, and New Year’s Eve alone. Many continued to avoid shopping in stores or being in crowds. This decision to remain separated from family and friends amplified their ongoing sense of isolation. Six months into the pandemic, Participant #15 said, “We’re just tired. I want to be able to go out to dinner. I want to be able to see my grandbabies.”

#### Theme two: professional image

They recalled the fear they felt six months ago. Now, protocols based on experience and research gave them confidence.

It’s more settled now. We have a better system. It’s become part of our normal daily stuff. There’s more known about it, so it’s not so scary. (Participant #6)

The novice nurses who felt unprepared for the first surge said they grew up quickly and now felt like veterans. Participant #20 recalled, “I have my own patients with COVID-19. I’m not just supporting the ICU nurses.”

But their overall sense of satisfaction with their clinical work declined.

When you can give the care that you want to give, you feel better about your profession. Early on, you think, Wait a minute. I’m better than this. I’m a better nurse. (Participant #15)

#### Theme three: communication

The staff continued their daily “huddle” meetings and noticed a difference from the early pandemic gatherings. Now they looked at problems like a team. Physicians, nurses, assistants, clerks were part of the solution. The nurses appreciated clear communication with their leadership.

Daily scheduling remained a frustrating aspect of working. Participant #20

It’s a lot not knowing where you’re going to be when you come into work. I’m texting other nurses ‘Where am I going? I hope I find a familiar face to help.

Connecting with patient’s families was primarily through Zoom. This compassionate measure could add to the nurses’ daily stress. As participant #35 said,

You had families watching 24 h. We made some rules–phone or Zoom from noon to 8 PM. Sometimes the families didn’t realize how very sick the patient was.

Reassurance was a common response to families’ concerns. As Participant #12 who worked the night shift recalled saying to relatives,

“I’m going to be here with your loved one so that you can sleep.”

#### Theme four: community reaction

The local community stayed supportive. Donated food continued to arrive. ‘Thank you’ signs remained on front lawns but, the earlier support from the March-April 2020 surge fractured as vaccinations and masks became political statements. This created a sense of disquiet among the staff. Participant #21 reacted to this change

People who don’t believe that it’s real, I want to say, ‘Come to the hospital. Go into a COVID-19 room, take care of that patient without a mask on and tell me that you feel comfortable. Or go wrap the body of a patient who died from COVID-19, without anything on, and tell me how you feel.

In the end, Participant #27 said,

“It’s frustrating because we’re going to be the ones taking care of them.”

#### Theme five: finding an emotional balance

Six months into the pandemic, they were physically and emotionally exhausted but all 35 nurses who remained in the study continued their bedside care. Their fears for themselves or their family getting sick diminished as knowledge about the virus and vaccines increased. They also felt they had the skills now to better care for patients. This combination made them feel more secure and confident in going to work every day.

Subjects would use the word “compartmentalize” to describe how they remained emotionally steady. “Anything non-work related, I deal with outside of work, and work related, I keep at work.” (Participant #9).

Some nurses stopped working overtime so they could have two consecutive days off and get a good night’s sleep. Continuing to meditate helped as did taking walks to lower their stress. A few found comfort in praying; others registered for graduate school. Their co-worker’s friendships remained their most effective ballast for support and balance. In the fall 2020, a group of nurses decided to support one another by building a garden. At the beginning of the pandemic, a decommissioned hospital on the medical center grounds became the hospice for COVID-19 patients. Nurses from closed units helped staff the new building. As COVID-19 mortality rates decreased, the hospice building was no longer needed. Nurses, who were delighted with this progress, also felt a sense of sadness. A staff nurse spoke with the hospice director and social worker and asked if they could honor all 152 patients who had been in the hospice facility, 94% of whom died [17]. The care they had given felt unfinished. They wanted one last gesture of care, so they built a memory garden. Nursing leaders provided a budget; the hospital engineers bought flowers, the nursing director and social worker purchased gloves and spades and invited the staff to create a garden on the traffic island at the entrance to the hospice facility. One plant for each patient. About fifty staff participated on two Saturdays. Wearing their scrubs, they dug holes and planted pink, white, yellow, orange, and blue pansies; posed for a group photo, listened to brief speeches, then put their arms around one another and cried. They named the traffic island, The Garden of Souls.

### Phase three findings

In the final phase, which ran from April through August 2021, we conducted interviews with the same 35 RNs who participated in the first and second phases. During this phase, the COVID-19 census fluctuated from a low of 16 patients and two deaths from mid-May through mid-June to a high of 125 patients with 19 deaths in mid-August during the Delta COVID-19 surge, per internal tracking and communications. Once nurses were no longer needed to care for COVID-19 patients, they returned to the O.R. and other specialty units and found their patients often let their diabetic or blood pressure care lapse. Still, returning to familiar units and patients was a comfort.

#### Theme one: exposure risk

When asked about their worries of getting sick, Participant #19 summed up the thoughts of others, “I’m not fearful anymore because I know what to do.”

After months of caring for COVID-19 patients they felt confident in their ability to work safely, “It’s part of the day to day. It’s a norm,” said Participant #17.

#### Theme two: professional image

The virus changed the nurses’ routines. Rather than going into rooms to administer a medication and returning to bathe a patient, they clustered their care to work more efficiently to complete both tasks together. The novice nurses felt comfortable having those difficult talks with families.

These nurses discovered new skills: They could wear masks and face shields and still have a conversation. Pediatric nurses learned to work with adult patients. Medications could be safely delivered from outside patient rooms. Participant #22 reflected others sentiment:

I’m not going to cherish this time, but I’m proud of the work we did.

#### Theme three: communication

As vaccination rates among the staff and the public increased, the visitation rules loosened to allow two visitors per patient from 12:00–8 P.M. This change made most families less anxious. Other families wanted permission for more than two visitors; others did not want to wear masks because they were vaccinated. With those families, nurses now had to explain the restrictions to people who didn’t want to cooperate.

Nurses began to spend time with their families. They felt more comfortable visiting their parents, letting their grandchildren swim in their backyard pools, planning a vacation.

#### Theme four: community reaction

Their thoughts about the community response remained mixed. The food deliveries, shoe donations and other gifts continued to help morale. Still, the subjects recognized that at this point in the pandemic, little could be done to change people’s minds about masks and vaccinations.

No matter what you tell them, they’re going to believe what they believe Participant #38.

Participant #41 was more frustrated at some of the public reaction.

When you watch others die, gasping for breath, wearing a mask is nothing.

#### Theme five: finding an emotional balance

After a year or more, these 35 nurses had figured out how to keep their balance and report for their shifts. Participant #43 realized,

I changed my attitude and now I take a lot better care of myself. If I’m having a stressful day, I leave it at work.

Others returned to their favorite past times: running, taking photographs, writing in journals, praying the rosary, and reading. Some went to private or group therapy while others participated in the wellness programs at the hospital.

The friendships they formed with co-workers continued to be a major source of solace and balance. This camaraderie gave them a valuable perspective, as Participant #30 said:

I think we need to understand how to move on and not hold anger or resentment towards the world about COVID.

The strength of these friendships and their success at caring for their patients during this extraordinary pandemic made them think about future crises. Participant #27 said,

I would do it again as I would not want my colleagues to do it alone.

## Discussion

The two aims of the study were to examine nurses’ thoughts about caring for COVID-19 patients over 15 months and to explore why these professionals remained at their positions while other nurses departed. A small number (6 out of 41 subjects) of participants left the study after Phase 1. No subjects left the study in Phases Two or Three. We were not able to compare findings from those who left with those who remained. We focused instead on what the 36 nurses did to stay. Scholars have studied the intent to leave work positions during the pandemic. Across the globe, factors linked to this resolve included fear of COVID-19, job stress/workload, low resilience, burnout, working conditions, and organizational culture/teamwork [18–21]. Several quantitative studies indicated that job stress, low resilience and burnout were linked to nurses’ reported intent to leave [18–21]. In a descriptive correlational study, El-Ashry et al. noted that 200 critical care nurses working in Egypt, who reported moderate or higher levels of flexibility had less intention to leave their jobs [22]. In Iran, 300 nurses working in 2021 had a significant association between fear of COVID-19 and job stress and intention to leave (influence value 0.313) [20]. n a cross-sectional study in Turkey, Bingöl et al. also found a moderate positive correlation between nurses’ job stress and intention to leave [19]. These findings support the importance of nurse managers to be aware of job stress and strive to address stressful situations for clinical nurses. A nationwide survey of nurses working in the USA by the National Council for State Boards of Nursing (NCSBN) revealed that 100,000 nurses had left the workforce during the pandemic [8]. The 36 study participants remained at their positions. In a communication to one of the authors (cj), a director in human resources reported a 15% increase in resignations and retirements was observed from 2019 to 2021. What motivated those in the study to stay? During the interviews, they spoke about several institutional factors like supportive leaders and a systemwide program which included availability of mental health professionals, meditation rooms and other supports that conveyed the sense that their superiors cared about the nurses. During their off-duty hours, they had opportunities to pursue personal activities they knew should balance the stress of their work shifts: jogging or aerobics, praying the rosary, talking or being with family, and enrolling in graduate school. Responding to interview questions, subjects stated that what helped them most in staying at their positions was the coming together with other nurses and coworkers in formal and informal support groups. This camaraderie was not planned, people came together to talk, eat a meal, or sit down. Many nurses felt that their colleagues best understood their difficult world. “You can laugh, decompress and get a helping hand. You’re never going at it alone,” recalled Participant #9. In reviewing the interviews with nurses who served in the Vietnam War, similar thoughts about the benefits of these informal get togethers where friendship, humor, and food relieved some of the stress of work and living in a dangerous world. An Army Nurse Corps officer who served at the 95th Evacuations Hospital in South Vietnam in 1969-70 remembered,

I don’t think I’ve ever been as close to people as I was in Vietnam…I couldn’t have made it through that year without them [23] (p48).

The importance of camaraderie was also reported by nurse veterans during other military conflicts [24]. Ambrose et al. studied 22 individuals in the USA and found that a sense of duty and cohesion with team members resulted in greater resilience to workplace stress [25]. These researchers found that the connection among healthcare staff went beyond pre-pandemic interactions. Phillips and colleagues studied 38 ED physicians and nurses in Oregon USA who participated in focus groups and spoke about communication and teamwork [26]. Their responses indicated that group meetings resulted in proactivity and openness.

At the medical center in this study, each weekday around 10 AM and 7PM, and noon on weekends, staff and managers from every hospital unit gathered for a meeting called a “huddle.” Updated information was shared by managers with staff on each unit at 7 pm information huddles. They discussed safety concerns and changes in policies and procedures. Physicians and managers reported what they learned at other meetings. “I always felt that I knew what was going on,” said a nurse (Participant #29) who could also check her assignment and patient records during those meetings.

The themes identified in this research provide knowledge and insight of nurses’ perceptions while caring for patients in unprecedented challenges, such as disasters or a pandemic. A summary of themes, definitions, clinical nurses’ perspectives, proposed leadership interventions and supporting research [27–39] are provided in detail [see Additional file 3]. The community help, the organizational support, personal networks, and the nurses’ personal resources provided a basis for them to remain at their positions. Another reason for their professional dependability seemed innate to their personalities. Classic German philosopher Immanuel Kant theorized how people react to a crisis, and his work remains relevant today [36]. At the moment of decision, individuals decide on a moral action which has three parts. The first moment of decision is what Kant emphasized, not the action or the consequences of that action. Individuals, like the nurses in this study, decided to act on the principle that working during COVID-19 was something they ought to do and what anyone in this circumstance with their skills should do. Participants #40 reply to an interview question illustrates Kant’s philosophy: “I felt like I had an obligation, I was scared. I almost called out sick at six o’clock in the morning. Then I’m like, ‘Just go.’ Everybody else is doing it. You’ll figure it out.”

Kant wondered why people had the capacity to look beyond a natural inclination to protect themselves [36]. Duty was the intention behind an individual’s decision to continue to work by focusing on something more than themselves. These nurses went to work even when they knew many patients would die no matter what they did. They felt they had to participate in the communal challenge of keeping COVID-19 under as much control as possible.

This sentiment of duty has been reported in Intensive Care Unit (ICU) nurses by Watson as well as by Ampos who studied nurses in Brazil [31, 33]. The idea of duty has been fundamental to the profession since Florence Nightingale’s era [37]. She wrote that a trained nurse should be a woman who looked beyond herself and cared for her patients. Both visionaries emphasized setting aside personal considerations when deciding on an action. Kant expanded on this idea with the belief that individuals who resolve that working is something they ought to do also is something that anyone in these circumstances should do. Participant #23 alluded to this idea of duty:

I don’t want to say I was forced to take care of adult COVID patients. But when all was happening, I looked at the bigger picture, and I thought, okay, they’re in need. Of course, I’m also going to help my peers in the ICU. I would do that today if this were to all start again.

## Limitations

Limitations to the study reflect restraints found in other longitudinal studies: the generalizability of the findings. A global pandemic such as COVID-19 where hundreds of thousands of nurses worked will likely result in multiple experiences and conclusions. A comparison of the six participants who did not complete all three interviews to those who did complete the study warrants additional research. An exploration of such variables as age, years of experience, race, ethnicity, shift, and specialty area may provide additional insight to retention.

## Conclusions

At the conclusion of this study, these nurses were still caring for COVID-19 patients. Their sense of COVID-19 as a time-limited crisis was over; these patients would be part of their daily assignments. As nurses who cared for hundreds of viral patients, they experienced humanity at its best and worst: anger, sadness, loss, as well as endurance and kindness. Reflecting on their lives from that time, the thinking behind their decision to work reflects Kant’s ideas of duty.

Planning for future disasters is imprecise. Experts can develop organizational, and community support learned during the COVID-19 pandemic to assist nurses and other health care staff. Leadership support, including but not limited to decreasing exposure risk, provision of appropriate clinical education, facilitation frequent communication, and fostering community and emotional support was identified as an important factor to nurses in this study. Support by managers, plus the nurse’s personal belief in the greater good over personal needs and desires could be foundational. While the nurses speculated about why they made the decisions they did, the knowledge that this combination of external and personal assets should provide support for nurses and other health care workers to endure.

## Supplementary Information

Below is the link to the electronic supplementary material.

Supplementary Material 1

Supplementary Material 2

Supplementary Material 3

## Data Availability

The data that support the findings of this study are available on reasonable request from the corresponding author. The data are not publicly available due to confidentiality or ethical restrictions.

## Abbreviations

AIDS: Acquired immunodeficiency syndrome
ED: Emergency Department
ICA: Intercoder agreement
ICU: Intensive care unit (first seen on page 17 as part of quote; defined on page 25)
IV: Intravenous (used as a quote not defined in text)
MICU: Medical Intensive Care Unit
PPE: Personal Protective Equipment
Psi: Parts per square inch (used as quote– not defined in text)
RN: Registered Nurse
USA: United States of America
