# Supplementary material for: A longitudinal qualitative study of clinical nurses caring for hospitalized adults during the first fifteen months of COVID-19: lessons in professional survival and leadership
Source: BMC Nurs. 2025 Aug 18;24:1079. doi: 10.1186/s12912-025-03628-2 (PMC12359894; doi:10.1186/s12912-025-03628-2)
Supplement: Supplementary file 1 — Supplementary Material 1 [file 12912_2025_3628_MOESM1_ESM.docx]

**Additional file 1: Interview Schedule (English)**

Structured discussion questions were prepared to initiate and guide the conversation. Clarification or requests for additional details about unanticipated topics related to the lived experience of caring for adults with COVID-19 were allowed to be incorporated during the study conversation. Clarifications and exploration were approved by the Institutional Review Board.

Discussion questions included an introduction to inform the participant that the interviewer wants to hear their story:

What was the professional image you had of yourself before COVID-19 pandemic?

1. What changed for you?
2. During the COVID-19 pandemic, if you could not be the nurse you had been: How did you deal that.

- [If clarification is needed: What about communication? Patients, their family members, other team members (staff), managers or leaders at the medical Center. Lack of knowledge about COVID-19?]

1. Please tell me the story of the worst clinical day you experienced. What was your reaction to that day?
2. Now, tell me a story of your best or most rewarding day. What was your reaction?
3. If I were to ask you about the topic of balance during your work, would you say that you have gotten your balance? How did you get your balance? What, if anything, helped bring you closer to balance?
4. What are you doing to prepare mentally for the next month? (Can you prepare?)
5. What are you going to do with your memories? [Explore if the participant guesses that the memories will go away soon?]
6. Suppose a year from now, if the virus has abated, you are asked by a group of nursing students about your clinical COVID-19 experience. What would you say?

Structure interview questions for sessions at three months and six months were based on responses and themes identified from the initial findings.

Potential questions at three and six months may include:

1. The last time we spoke about your perception of you as a nurse [researcher may provide a brief summary of the prior discussion]. Can you tell me, today, about your perception of you as a nurse? Has it changed since we last spoke (three or six months ago)?
2. Tell me about your communication with patients, family members, team members (staff), and managers/leaders.
3. Tell me a story about caring for a patient.

- Tell me about a challenging day.
- Tell me about a meaningful or rewarding experience.

1. Tell me about your balance.
2. Have you used any resources provided by this medical center or elsewhere to help you find balance?
3. Do you prepare mentally or emotionally for work? What specifically do you do? (Do you think you can prepare?)
4. If you think back to the start of the pandemic in March/April of 2020, tell me about your memories.
5. If a year from now, if the virus is abated, and you were asked by a group of nursing students about your clinical COVID-19 experience, what would you tell them?
6. Is there anything else you would like to share with me, that I did not specifically ask about?

**Second and Third Interviews**

For the second and third interviews the interviewers asked the participants questions before the start of the interview Do you have any questions? [Discuss questions] If any questions (or other questions) arise at any point in this study, you can feel free to ask them at any time. I would be more than happy to answer your questions.

1. The last time we spoke about your perception of you as a nurse [The researcher provides a brief summary of the prior discussion, to serve as member checking. Notations of clarification or modifications are noted.]. Is this accurate? Do you have any clarifications or anything additional you would like to share?

 Can you tell me, today, about your perception of you as a nurse?

Probe: Has your perception of you self as a nurse changed since we last spoke (three months ago)?  Since the start of the study (approximately six months ago)?

2. Tell me about your communication with patients, family members, team members (staff), and managers/leaders.

3. Tell me a story about caring for a patient.

Probe: Tell me about a challenging day.

1. Tell me about your balance.
2. Have you used any resources provided by this medical center or elsewhere to help you find balance?
3. Do you prepare mentally or emotionally for work? What specifically do you do?

Probe: Do you think you can prepare?

1. If you think back to the start of the pandemic in March/April of 2020, tell me about your memories.
2. If a year from now, if the virus is abated, and you were asked by a group of nursing students about your clinical COVID-19 experience, what would you tell them?

What would you say to the students?

1. Is there anything else you would like to share with me, that I did not specifically ask about?

At the end of each interview, each participant was reminded of resources: *Just a reminder that resources are available to you in case you need them. The resources may be used if you need help to process what has been discussed here. Thank you for taking time to participate today.*

**Additional Question for the Third Interviews**

1. Did you approach the second phase of the pandemic differently? If so, what was different?
2. We continue to be cautious, wear masks, wash hands and maintain social distance. Not everyone is abiding by these precautions. What are your thoughts or feelings about that?

In closing at the final interview, interviewers thank the participant. The interviewers share information about the dissemination of the study by saying something similar to: *I just want to remind you is that when we present this data, or when we write it up, we won’t use any names. So we won’t be able to give you credit for the wonderful things that you shared with us. But I just want to thank you for meeting with me multiple times, for taking time, and importantly, for everything that you’ve done for patients, because it really made a difference.*
