# Supplementary material for: A longitudinal qualitative study of clinical nurses caring for hospitalized adults during the first fifteen months of COVID-19: lessons in professional survival and leadership
Source: BMC Nurs. 2025 Aug 18;24:1079. doi: 10.1186/s12912-025-03628-2 (PMC12359894; doi:10.1186/s12912-025-03628-2)
Supplement: Supplementary file 2 — Supplementary Material 2 [file 12912_2025_3628_MOESM2_ESM.docx]

| **Codes (42)*** | **Themes (5)** |
| --- | --- |
|  |  |
| Exposure Risk | Exposure Risk |
| Emotion |  |
| Finding your balance |  |
| Meaningful quotes |  |
| Worst clinical day story |  |
| Best clinical day story |  |
| Communication patient’s family | Communication |
| Communication managers/leaders |  |
| Communication team |  |
| Communication patient |  |
| Communication nurse family/friends |  |
| Memories |  |
| Camaraderie |  |
| Professional image prior | Professional image |
| Professional self, adapting to change |  |
| Professional self-image changes |  |
| Professional image changes (Second interview) |  |
| Professional image changes (Third interview) |  |
| Professional self-image changes – no change |  |
| Preparing for next month/next wave |  |
| Advice to future nurses after pandemic |  |
| Competent level of care |  |
| Thinking on worst day |  |
| Thinking on best day |  |
| Searching for balance | Finding an emotional balance |
| Best clinical day story |  |
| Historical reference |  |
| Compassion |  |
| Vigilance no preparation |  |
| Exposure |  |
| Military reference |  |
| Shared experience |  |
| Disbelief |  |
| Community support | Community reaction |
| Community negative |  |
| Novice nurse |  |
| Trigger |  |
| Spirituality, God, beliefs, religion, prayer |  |
| Spiritual, religious beliefs, God referenced |  |
| Member check do not agree |  |
| No best day |  |
| No worst day |  |

*Codes were assigned by researchers independently, therefore, similar wording was created and then merged.
